# Decreased Respiratory-Related Absenteeism among Preschool Students after Installation of Upper Room Germicidal Ultraviolet Light: Analysis of Newly Discovered Historical Data

**DOI:** 10.3390/ijerph20032536

**Published:** 2023-01-31

**Authors:** Christopher W. Ryan

**Affiliations:** 1 Binghamton Clinical Campus, SUNY Upstate Medical University, 48 Corliss Avenue, Johnson City, NY 13790, USA; cryan@binghamton.edu; 2 Broome County Health Department, 225 Front Street, Binghamton, NY 13905, USA

**Keywords:** germicidal ultraviolet light, upper room germicidal irradiation, preschool, absenteeism, respiratory infection

## Abstract

The COVID-19 pandemic has brought renewed urgency to air disinfection. Upper room germicidal ultraviolet light (GUV) disinfects room air very efficiently. Its effect on practical outcomes in public settings remains unclear, but history may provide some insights. An interrupted time series model was fitted to a newly discovered dataset of attendance records from a preschool between 1941 to 1949, where GUV was installed in December 1945. GUV was associated with a sizable reduction in child absenteeism due to respiratory illnesses of any cause. Odds ratios for the effect ranged from 0.5 to 0.77, depending on the season. In all but high summer, model-predicted absenteeism rates were reduced by between a third and a half by GUV. Wider use of upper room germicidal UV systems in schools and preschools may be worthwhile, to reduce absenteeism due to respiratory illness and the educational, social, and economic consequences that ensue.

## 1. Introduction

The COVID-19 pandemic has brought renewed urgency to the problem of indoor air disinfection. Ultraviolet-C (UV-C) radiation inactivates micro-organisms in the air, in both benchtop [1,2] and room-sized [3,4,5] chambers. Fixtures that emit UV-C at a wavelength of 254 nm were developed in the 1930s. Professionally designed and installed 254 nm UV fixtures are hung high on walls, where they narrowly collimate their output to a layer just below the ceiling, well above the heads of the room occupants. This capitalizes on the vertical convection currents that are present in most occupied rooms. As air rises to the ceiling, it is disinfected by the UV-C light and then re-circulates back down to the occupied zone. (Hereafter, these systems will be referred to as upper room GUV or simply GUV). Professionally designed and installed systems do not expose room occupants to any meaningful amount of UV-C [6,7]. UV-C does not cause skin cancer [8].

The control of respiratory infections is particularly important, and also particularly challenging, in indoor congregate settings such as schools and preschools. Beyond their vital role in the educational, emotional, and social development of children, schools are woven into the socioeconomic fabric of society and serve as social supports for students and families. Most schools in the United States (US) closed to in-person instruction in the spring of 2020, in an attempt to mitigate the emerging COVID-19 pandemic. The consequences of missing school fell most heavily upon vulnerable families, exacerbating inequities [9,10,11]. Absenteeism interferes with parental work activities that generate household income [12,13]. The difficulty in operating hospitals during a pandemic, when school closures left many staff members unable to work for lack of childcare, was ironic and poignant [14].

When they reopened in the fall of 2020, US schools emphasized masking, frequent testing, and eventually vaccination. These infection control measures involved substantial individual behavior change, often mandated, and were not always well-received by students, parents or staff. Relying on individual behavior change generally yields the least health impact while demanding the most effort [15]. By contrast, engineering interventions that require no individual effort yield the greatest population health benefits, and are more efficient. GUV is an engineering intervention that may be useful.

Early studies of the effect of GUV in school settings on the incidence of respiratory infections of specific etiologies yielded mixed findings [16,17,18]. Experimental designs differed, observation periods were short, and analyses were limited by the methods available at the time. A few years later, Gelperin and colleagues in New Haven, Connecticut installed GUV in some classrooms in each of 8 out of 36 buildings in the month of February. They concluded there was no discernible effect on absenteeism rates due to respiratory illnesses, but again, the observation period was only 4.5 months [19]. More recently, Su et al. found no difference in absenteeism rates, in a single elementary school, between students in two classrooms with GUV and those in four classrooms without. However, their primary interest was the comparative efficiency of two different air sampling techniques, not absenteeism, and the study had little power to detect differences in the latter [20].

To truly understand the utility of GUV in the control of respiratory illness in schools, a prospective randomized trial using modern methods would be ideal, but such a study would face many design, analysis, logistical, and even political hurdles. Meanwhile, history may provide another window into the question. Although rarely found in general public settings in the US today, GUV was used in that manner for a time in the 1940s to early 1950s. That cyclical history has been reviewed by Reed [21]. The serendipitous discovery of several historical documents in the archives of a major current-day manufacturer of GUV systems presented an opportunity to assess the practical effect of upper room GUV on student absenteeism in a preschool over a span of 9 years.

## 2. Materials and Methods

### 2.1. The Source Document and Data

The documents were shared via mail by Ann Wysocki, Director of Marketing at Atlantic Ultraviolet. She discovered them in a single folder in the company’s archives. Atlantic Ultraviolet was founded in the early 1960s and was the sole east coast distributor for Westinghouse’s germicidal ultraviolet light bulbs. In the late 1960s, they distanced themselves somewhat from Westinghouse and began manufacturing their own ultraviolet units (personal communication, Ann Wysocki, Atlantic Ultraviolet Corporation).

While the documents in the folder all pertain to germicidal ultraviolet light, the nature of the documents varies. The folder contains:

- Several documents that are likely internal company documents generated by GUV manufacturers or vendors, in particular Westinghouse (makers of a GUV lamp called “Sterilamp”) and Sanitron, a predecessor of Atlantic Ultraviolet. These corporate documents are identifiable either by the Westinghouse Lamp Division letterhead, or the appearance of a small “ASC-nnn” notation, where “nnn” is a number. This appears to be a notation used by Westinghouse Electric Corporation on some of its internal documents, as seen, for example, in the reference lists in a 1966 article about GUV by Minkin [22], and in a 1971 US Government Printing Office publication authored by Dubin–Mindell–Bloome Associates [23].
- Photocopies of short New York Times articles, labelled May 24 and 28, 1950, about GUV deployments and trials.
- A photocopy of an article presented at the 140th annual meeting of the Medical Society of the State of New York, section on pediatrics, on 2 May 1946, and published in the New York State Journal of Medicine [24].

Only some of the documents contain an author’s name. Of those documents that are dated, the dates range from 1946 to 1950.

The document of interest for the present study is entitled “Reduction in absentee rate at the [Preschool A] using Westinghouse germicidal Sterilamps”. The document displays no author or affiliation but carries the notation “ASC 149”, so it is likely to be a publication of Westinghouse Electric Corporation. Preschool A is located in what was at the time a moderately large Mid-Atlantic US city. Although Preschool A is still in operation, little is known about how it functioned 70 years ago. The document contains only one detail: that in the summer months, the children spent a lot of time outdoors. Current staff at Preschool A have related that the GUV fixtures are no longer present, and they did not have personal knowledge of their previous installation, use, or removal (personal communication from a Preschool A administrator).

The document includes a table of attendance data from January 1941 to November 1949, inclusive. Recorded are the number of possible/available child-days of attendance, and the number of child-days missed due to respiratory illness, for each age group each month. The nature or etiology of the respiratory illnesses causing the absences is not described in the source document. There are four age groups: ages 2, 3, 4, and 5. The 5-year-olds were considered to be in a kindergarten program that did not operate in July or August. For the younger age groups, attendance figures are, with rare exceptions, recorded year-round. “Sterilamp” GUV systems were installed in all classrooms in December 1945. The document does not explain why, but other historical documents indicate Preschool A often participated in research studies. Analytically, the document contains just one simple graph (reproduced in the Supplementary Materials), which is highly suggestive of a reduction of absenteeism associated with the presence of GUV. However, there was no attempt to fit a statistical model, account for serial autocorrelation, conduct hypothesis tests, or construct confidence intervals.

An interrupted times series model was fit to these attendance data, in an effort to measure the effect of the upper room GUV systems on student absenteeism.

### 2.2. Statistical Analysis

Without knowing how much the different age groups interacted, the data were pooled across the age groups, and absenteeism was analyzed in a school-wide fashion. Monthly absenteeism rates were calculated by dividing the number of child-days missed by the number of child-days available for each month, yielding a proportion of available child-days missed in each month. The logit (log-odds) transformation of those absenteeism rates was used as the response in the interrupted time series model, while assuming an autoregressive lag-1 (AR(1)) correlation structure between the monthly observations. The predictor of interest was the presence/absence of upper room GUV. As the GUV fixtures were installed sometime during December 1945, January 1946 was considered the first month with GUV.

Absenteeism due to respiratory illnesses, and the effect upon it of GUV, if any, could reasonably be expected to vary seasonally, due to seasonal fluctuations in (1) overall respiratory disease incidence, and (2) the prevailing location of school activities—more confined indoor spaces when the weather was generally colder versus more spacious and aerated outdoor areas when it was generally more pleasant. As a proxy for both these cyclical phenomena, each month’s average temperature was included as a predictor in the model. The temperature data, from January 1941 to November 1949 inclusive, were obtained from the National Weather Service: https://www.weather.gov/wrh/Climate?wfo=lwx, accessed on 8 July 2022. The temperatures were converted to Celsius degrees.

Confounding between the passage of time and the effect of an intervention instituted at a specific point in time is challenging. Effects on the response that appear to be associated with the intervention may, at least in part, be due to secular trends or unmeasured temporal factors. To determine whether there were linear temporal trends in absenteeism unrelated to any putative effect of GUV, initially terms for two piecewise linear temporal trends—one before GUV was installed, and one after—were included as predictors, with time measured as the number of months elapsed since the start of the time series in January 1941.

To assess for underlying trends in absenteeism before and after GUV but unrelated to its installation, a two-sided simultaneous hypothesis test, using Hommel’s method of multiplicity adjustment, Ref. [25] was used to test those terms in the initial model. Using the final model, odds ratios for the reduction in absenteeism in the presence of GUV, at the mean monthly temperature in each of the four seasons, were calculated. Given the mechanisms of the action of GUV—cross-linking nucleotides in nucleic acids, and interfering with transcription and replication—there is no plausible biological or physical mechanism whereby GUV could increase the concentration of airborne pathogens, and thus the incidence of respiratory illness and the ensuing absenteeism. As that possibility did not warrant consideration, one-sided simultaneous confidence intervals around the four odds ratios were constructed. (For completeness, two-sided confidence intervals for these odds ratios were also constructed and are included in the Supplementary Materials).

The predicted rates of monthly absenteeism, with and without GUV, and across the range of observed temperatures, were calculated and plotted, along with their point-wise 95% confidence intervals.

R version 4.2.0 (R Foundation) was used for the analysis. The R code, and a pipe-delimited plain-text file containing the complete data, are both available in the Supplementary Materials.

### 2.3. Reviews and Approvals

The SUNY Upstate Medical University Institutional Review Board determined that this project, number 1914921-1, was not human subject research, because it relied only on aggregated, non-identifiable data.

## 3. Results

### 3.1. Data Exploration

After pooling across the age groups, the analytical dataset comprised 107 monthly observations. There were 59 months without upper room GUV, followed by 48 with GUV. Monthly respiratory-related absenteeism rates ranged from 1.7% to 37.5%, with a median of 8.7%. Monthly average temperatures ranged from −1.8 C to 27.2 C, with a median of 14.3 C. Temperatures varied seasonally, as expected.

**Figure 1:**
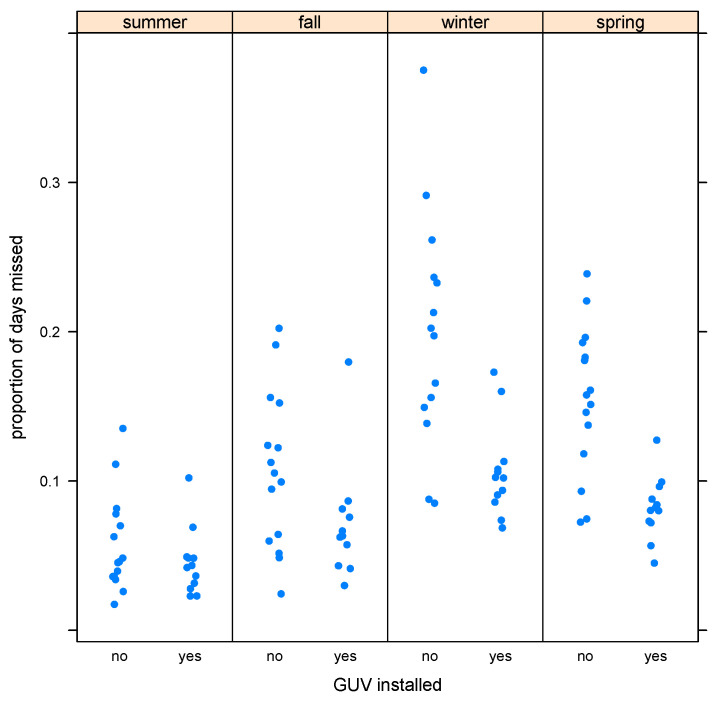
Monthly absenteeism rates, grouped by season.

**Figure 2:**
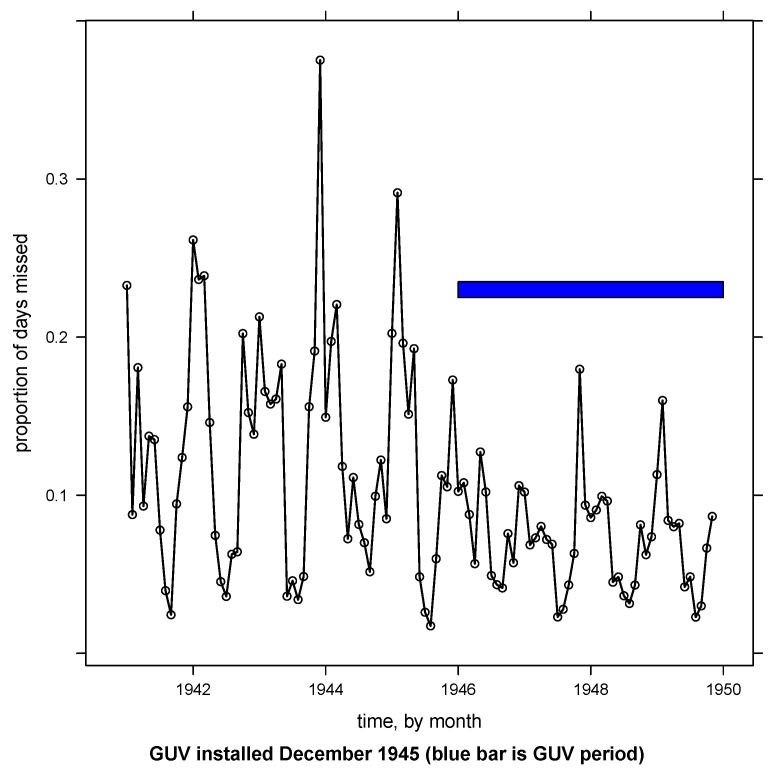
Monthly absenteeism rate for all age groups combined. The blue bar indicates the GUV period.

Observed extremes of absenteeism were more marked in the winter months, and these extremes may have been blunted during the GUV years, resulting in less apparent variation in absenteeism rates during the GUV period (Figure 1 and Figure 2).

### 3.2. Modeling

Equation (1) shows the initial, full model considered. Initial modeling and graphical diagnostics indicated that the effect of temperature on absenteeism might be quadratic, so the square of the average monthly temperature was included as a predictor, along with its interaction with GUV. Details of several models are shown in the Supplementary Materials.

(1) $$\begin{array}{cc} \log\frac{P_{t}}{1-P_{t}} & =\;\beta_{1}\;+\;\beta_{2}\left(GUV_{t}\right)\;+\;\beta_{3}\left(temperature_{t}\right)\;+\;\beta_{4}\left(temperature_{t}^{2}\right)+\\ \; & \;\beta_{5}\left(GUV_{t}\right)\left(temperature_{t}\right)\;+\;\beta_{6}\left(GUV_{t}\right)\left(temperature_{t}^{2}\right)+\\ \; & \;\beta_{7}\left(t\right)\;+\;\beta_{8}\left(t\right)\left(GUV_{t}\right)+\nu_{t} \end{array}$$

where

$$\begin{array}{cc} \nu_{t} & \;=\;\rho \nu_{t-1}\;+\;\epsilon_{t}\;\mathrm{and}\;\epsilon_{t}\;∼\;N\left(0,\sigma_{\epsilon }^{2}\right)\\ t & \;=\;\mathrm{month},\;\mathrm{numbered}\;\mathrm{sequentially}.\;\mathrm{GUV}\;\mathrm{installed}\;\mathrm{at}\;t\;=\;60\\ P_{t} & \;=\;\mathrm{proportion}\;\mathrm{of}\;\mathrm{possible}\;\mathrm{student-days}\;\mathrm{missed}\;\mathrm{in}\;\mathrm{month}t\\ GUV_{t} & \;=\;\mathrm{GUV}\;\mathrm{present}\;(1)\;\mathrm{or}\;\mathrm{absent}\;(0)\;\mathrm{in}\;\mathrm{month}\;t\\ {\mathrm{temperature}}_{t} & \;=\;\mathrm{mean}\;\mathrm{temperature}\;\mathrm{in}\;\mathrm{month}\;t \end{array}$$

Whether any time trends were needed in the model was a critical question. In the model represented by Equation (1), the coefficient on the time index term ($\beta_{7}$) represents the linear effect of time before the installation of GUV. The sum of $\beta_{7}$ and the coefficient on the guv:time interaction term ($\beta_{8}$) represents the linear effect of time after the installation of GUV. After fitting the initial model (Equation (1)) to the data, a two-sided test failed to reject, at the 0.05 level, the null hypothesis that these two time trends were simultaneously zero (*p*-values 0.45 and 0.45 for the pre-GUV and post-GUV time trends, respectively). Qualitatively, this comports with Figure 2. Therefore, all time-related terms were removed from the mean structure of the model, but the AR(1) autocorrelation structure was retained, leaving the model in Equation (2) as the working model. This is Model (4) in the Supplementary Materials, and it will be the basis for all interpretation and diagnostics henceforth.

(2) $$\begin{array}{cc} \log\frac{P_{t}}{1-P_{t}} & =\beta_{1}+\beta_{2}\left(GUV_{t}\right)+\beta_{3}\left(temperature_{t}\right)+\beta_{4}\left(temperature_{t}^{2}\right)+\\ \; & \;\beta_{5}\left(GUV_{t}\right)\left(temperature_{t}\right)+\beta_{6}\left(GUV_{t}\right)\left(temperature_{t}^{2}\right)+\nu_{t} \end{array}$$

where notation is the same as in Equation (1).

A likelihood ratio test of the working model in Equation (2) against the same model with all GUV-related terms removed yielded a $\chi^{2}$ test statistic of 18.35 on 2 degrees of freedom, for a *p*-value less than 0.00011, suggesting that GUV played a meaningful role in the model.

Fitted to data, the working model yielded the following results, estimating predicted monthly absenteeism:

(3) $$\begin{array}{cc} \hat{\log\frac{P_{t}}{1-P_{t}}} & =-1.509+-0.674\left(GUV_{t}\right)+0.02\left(temperature_{t}\right)+-0.003\left(temperature_{t}^{2}\right)+\\ \; & \;-0.012\left(GUV_{t}\right)\left(temperature_{t}\right)+0.001\left(GUV_{t}\right)\left(temperature_{t}^{2}\right) \end{array}$$

Diagnostic plots for the working model, shown in the Supplementary Materials, were generally reassuring, except for some remaining autocorrelation and seasonality, especially at a lag of around 4 months.

### 3.3. Interpretation of the Working Model

**Figure 3:**
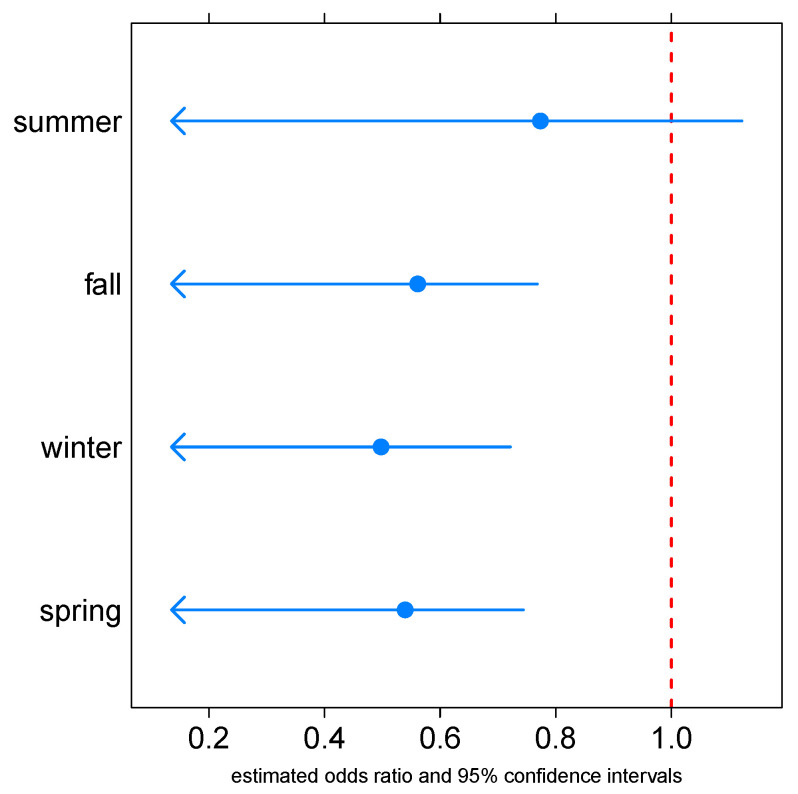
Odds ratios and their one-sided 95% confidence intervals for reduction in any-respiratory-cause preschool absenteeism associated with upper room germicidal light, at four illustrative temperatures—the mean temperature of each of the four seasons.

As the working model allows for an interaction between temperature (as a proxy for seasonal respiratory epidemiology) and the presence of GUV, the effect of the latter is temperature-specific. Odds ratios, and their simultaneous one-sided 95% confidence intervals, for the reduction of absenteeism associated with GUV at illustrative temperatures (the mean temperature of each conventional 3-month season) are shown in Figure 3. At non-summer temperatures, the ORs were all approximately 0.5 to 0.6, and suggested a significant reduction in any-cause respiratory absenteeism. For completeness, a similar graph with two-sided confidence intervals is included in the Supplementary Materials.

**Figure 4:**
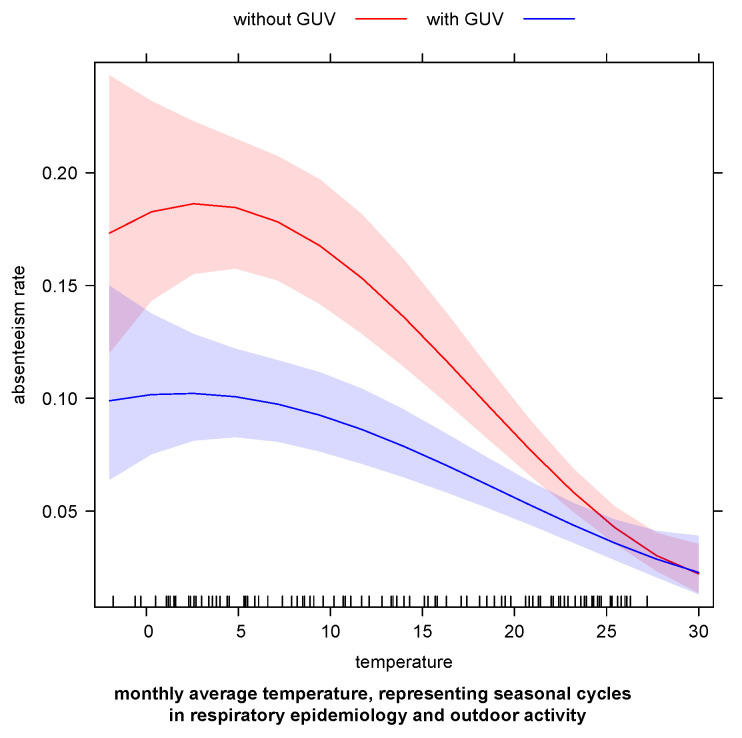
Solid lines show model-estimated monthly child absenteeism rates from preschool due to respiratory illness before and after upper room GUV was installed. Shaded regions are 95% pointwise confidence bands for each estimate. Red represents the months prior to GUV, while blue represents months with GUV. The effect varies by monthly average temperature (used as a proxy for seasonal cycles in respiratory epidemiology and in child outdoor activity), but GUV reduces predicted absenteeism by nearly half at all but the highest summer temperatures.

On the scale of absenteeism rates, Figure 4 shows the reduction in respiratory-related absenteeism in the presence of GUV, and how that effect varies with monthly average temperature. Save for peak summer temperatures, predicted absenteeism rates due to any-cause respiratory illness are significantly and meaningfully lower when upper room GUV is present.

**Figure 5:**
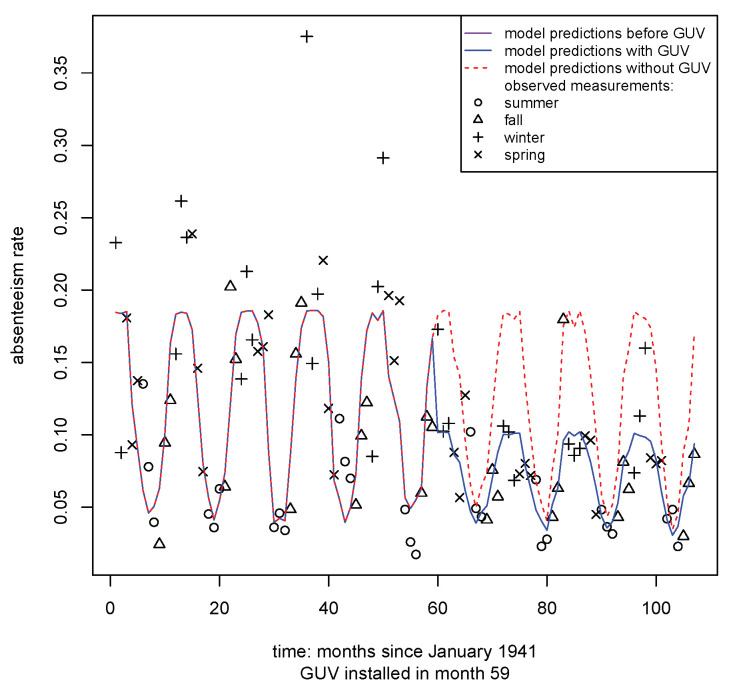
Time series of observed and model-predicted monthly absenteeism rates, and the model-predicted rates had GUV not been installed in December 1945 (dotted red line). The first complete month with GUV was January 1946 (month 60).

Figure 5 illustrates the observed and model-predicted monthly absenteeism rate, and the counterfactual: the model-predicted upper respiratory absenteeism rate had upper room GUV not been installed in December 1945.

## 4. Discussion

Previous studies of upper room GUV in schools have often focused on the incidence of specific respiratory diagnoses, such as measles, mumps, or chickenpox [16,17,18], but the effects on overall incidence of the diseases were not always clear. In some of the studies, there may have been a “blunted” epidemiologic pattern in settings with GUV: a lower incidence rate drawn out over a longer period of time [18]. Whether this would be valued by schools or families is unknown. Irrespective of specific diagnosis, however, all-cause absenteeism from preschool is a meaningful operational outcome measure of great concern to families, school administrators, and parents’ employers. Respiratory syndromes are one of the most common causes of school absenteeism. The present analysis demonstrated a sizable reduction in child absenteeism from preschool due to respiratory illnesses of any cause after the installation of upper room germicidal ultraviolet lights. In all but peak summer months, absenteeism rates with GUV were a third to a half of what they had been without it. The reduction appeared to be abrupt, concurrent with the installation of GUV systems in December 1945. There was no discernible downward temporal trend in absenteeism either before or after the installation.

In earlier studies, designs in which all classrooms in a building were outfitted with GUV, and a separate building was used as a control, yielded slightly more encouraging results in reducing disease incidence than those in which irradiated classrooms and control classrooms were present in the same building. Examples of the former design include Swarthmore public elementary school versus high school under Wells [16], and Cato–Meridian public schools versus Mexico public schools under Perkins and Bahlke [17,18]. The studies by Gelperin [19] and Su [20] represent the latter design, with “internal controls”, meaning some rooms in a school building were outfitted with GUV, while others in the same building were not. Over very short observation periods, neither of those investigators reported a difference in absenteeism associated with GUV. Given the inevitable interpersonal interactions between children and teachers throughout a school building, it is worth considering whether the effect of GUV may depend on whether all classrooms in a building are outfitted. The present analysis was of a whole-building GUV installation with a much longer observation period than any previous published work, and showed a meaningful reduction in absenteeism.

### Limitations

This report describes the attendance experience at a single preschool that installed upper room GUV. While the observation period was substantially longer than any other reported in the literature, and the reduction in absenteeism after installation was quite pronounced, the findings here may not generalize to other schools. As more schools install GUV in the present day, similar studies of their attendance experiences would be worthwhile.

Interrupted time series analyses are subject to a number of limitations. In the absence of a concurrent control group, it is impossible to say with certainty that the putative effect of the intervention was not due to some other factor or event that occurred around the same time. The occurrence of such a factor or event is particularly difficult to discern at a 70-year remove.

It is difficult to extrapolate from the overall epidemiologic environment of the 1940s to that of the present, but respiratory illnesses of a variety of causes are still common among schoolchildren. Furthermore, as the COVID-19 pandemic demonstrates, the human species remains vulnerable to pandemics of novel respiratory pathogens, to say nothing of the well-known annual influenza season.

The overall community incidence of respiratory illnesses in children may vary cyclically across years. It is possible that the early, non-GUV months in this time series were unusually “bad” in that regard, and that the decrease after the installation of GUV was just part of that natural cycle. It is possible that the installation was in fact motivated by perceived high levels of absenteeism in 1940–1945, a peak that was destined to fade over the following few years regardless. However, the historical record provides little support for the theory of particularly severe outbreaks of respiratory diseases during the pre-GUV years of 1941 to 1945. In fact, an influenza epidemic was reported in the 1946–1947 season [26,27,28], when GUV was already present in Preschool A, yet the absenteeism rate during that season remained significantly lower than in the preceding 5 years. The reason for the installation of GUV at Preschool A is unknown at present, but their decision-making process, for both the installation and the removal, would be an interesting topic for further research.

No information is available in the source document about how “absenteeism due to respiratory illness” was defined and counted, or by whom. No doubt a wide variety of medical conditions are subsumed under the syndrome of respiratory illness, not all of which are infectious or communicable. As any effect of GUV on reducing respiratory illness could only conceivably apply to the subset that is communicable, a measurable reduction in all-cause respiratory absenteeism in the presence of GUV is all the more notable.

The source document is also silent on: (1) any observed adverse effects attributable to the GUV; (2) the technical specifications of 1940s-era Sterilamps, which were likely different from those used today.

## 5. Conclusions

Air disinfection in indoor congregate settings remains a critical issue. This modern analysis of historical data demonstrated a significant and operationally meaningful reduction in absenteeism due to respiratory illness of any cause after upper room germicidal UV light fixtures were installed in a preschool. Wider use of GUV in schools and preschools may be worthwhile, to reduce absenteeism and its educational, social, and economic consequences.

## Data Availability

The absenteeism data analyzed in this study are available with the Supplementary Materials and at https://www.medrxiv.org/content/10.1101/2022.08.19.22278959v1.supplementary-material, last accessed on 8 January 2023.

## Supplementary Materials

The following supporting information can be downloaded at: https://www.mdpi.com/article/10.3390/ijerph20032536/s1. Figure S1: The single graph that appeared in the original document entitled “Reduction in absentee rate at the [pre-school A] using Westinghouse germicidal Sterilamps”.; Figure S2: Inclusion of a quadratic term (bottom) eliminates a pattern otherwise found in the residuals from the working model with respect to monthly average temperature (top).; Figure S3: Residuals versus fitted values show no particular pattern that would be concerning.; Figure S4: Top: There is slight heteroskedasticity in residuals between pre- and post-GUV periods. Middle: residuals by monthly average temperature show no particular pattern. Bottom: Time participates in the working model only in the serial autocorrelation structure. Residuals by time show no particular pattern.; Figure S5: Residuals are a reasonable fit to a Gaussian distribution. Top: qqnormal plot. Bottom: kernel density plot.; Figure S6: Some residual autocorrelation remains, especially at a lag of four months.; Figure S7: Two-sided odds ratios for effect of GUV on odds of absence, at four illustrative temperatures—the mean temperature for each season.; Table S1: Model comparison.

## Abbreviations

The following abbreviations are used in this manuscript:

GUV	upper room germicidal ultraviolet light
