# Supplementary material for: Decreased Respiratory-Related Absenteeism among Preschool Students after Installation of Upper Room Germicidal Ultraviolet Light: Analysis of Newly Discovered Historical Data
Source: Int J Environ Res Public Health. 2023 Jan 31;20(3):2536. doi: 10.3390/ijerph20032536 (PMC9915224; doi:10.3390/ijerph20032536)
Supplement: Supplementary file 1 [file ijerph-20-02536-s001.zip › ijerph-2080359-supplementary.pdf]

Supplemental Materials for "Decreased respiratory-related absenteeism among pre-school students after installation of upper-room germicidal ultraviolet light: analysis of newly discovered historical data"

Christopher W. Ryan, MD, MS, MSPH

January 25, 2023

Figure 1 is the single graph appearing in the original historical document (with the name of the preschool redacted).

Table 1 compares several models fit to the data. All models were fit via generalized least squares, allowing for different variances under pre-GUV and post-GUV periods, and with auto-regressive (1-month lag) correlation structure within pre- and post-GUV periods. As expected in an interventional time series analysis, the intervention (installation of GUV) is confounded with the passage of time. When GUV and time are both included (Model (1)), there is no appreciable temporal trend in absenteeism during either pre- or post-GUV periods. Therefore, on statistical and substantive grounds, Model (4), without terms for time, is considered the working model.

Figure 2 shows the need for a quadratic temperature term.

Figures 3 through 6 are diagnostic plots from the working model, Model (4) in Table 1.

Figure 7 shows two-sided odds ratios for effect of GUV on odds of absence, at four illustrative temperatures—the mean temperature for each season. These are little different from the one-sided intervals shown in the main manuscript.

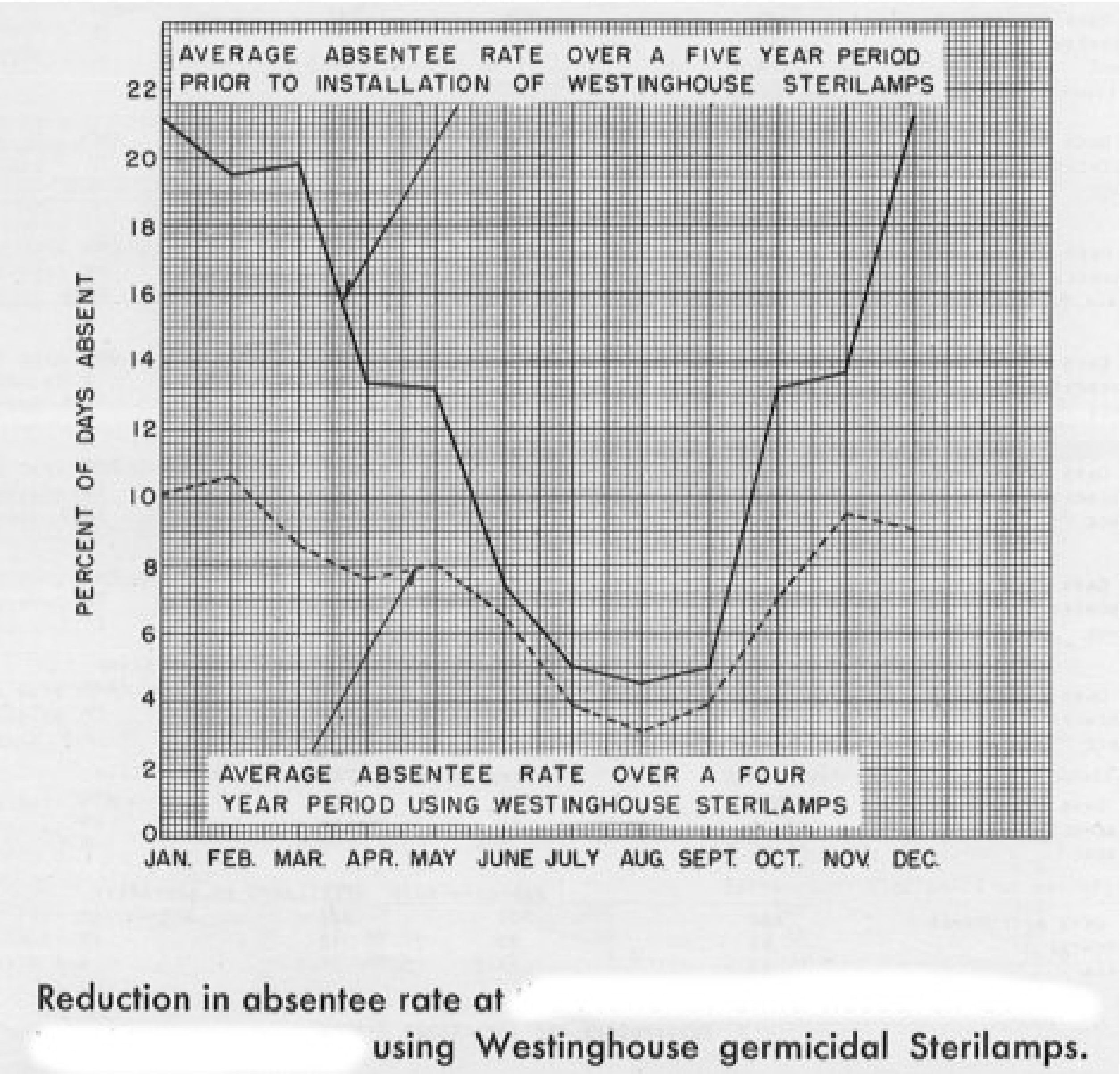

Figure 1: The single graph that appeared in the original document entitled "Reduction in absentee rate at the [pre-school A] using Westinghouse germicidal Sterilamps."

Table 1: Model comparison

	Dependent variable:					
	log-odds of monthly absenteeism rate					
	(1)	(2)	(3)	(4)	(5)	(6)
Constant	−1.432	−1.123	−1.403	−1.509	−1.338	−1.495
	(0.193)	(0.184)	(0.156)	(0.164)	(0.152)	(0.175)
guv.fac1	−0.303	−0.449		−0.674	−0.357	−0.228
	(0.504)	(0.538)		(0.246)	(0.480)	(0.500)
temperature	0.023	−0.066	0.021	0.020		0.018
	(0.029)	(0.008)	(0.022)	(0.029)		(0.021)
time.index	−0.003	−0.002	−0.008		−0.003	−0.003
	(0.004)	(0.004)	(0.002)		(0.004)	(0.004)
I(temperatureˆ2)	−0.003		−0.003	−0.003	−0.002	−0.003
	(0.001)		(0.001)	(0.001)	(0.0003)	(0.001)
guv.fac1:temperature	−0.010	0.022		−0.012		
	(0.042)	(0.012)		(0.042)		
guv.fac1:time.index	−0.003	−0.003			−0.003	−0.001
	(0.007)	(0.008)			(0.007)	(0.007)
guv.fac1:I(temperatureˆ2)	0.001			0.001	0.001	
	(0.002)			(0.002)	(0.0004)	
Observations	107	107	107	107	107	107
Log Likelihood	−54.050	−60.978	−57.255	−54.978	−54.490	−56.100
Akaike Inf. Crit.	128.099	137.955	126.511	125.955	124.981	128.200
Bayesian Inf. Crit.	154.827	159.338	142.548	147.338	146.364	149.582

Note:

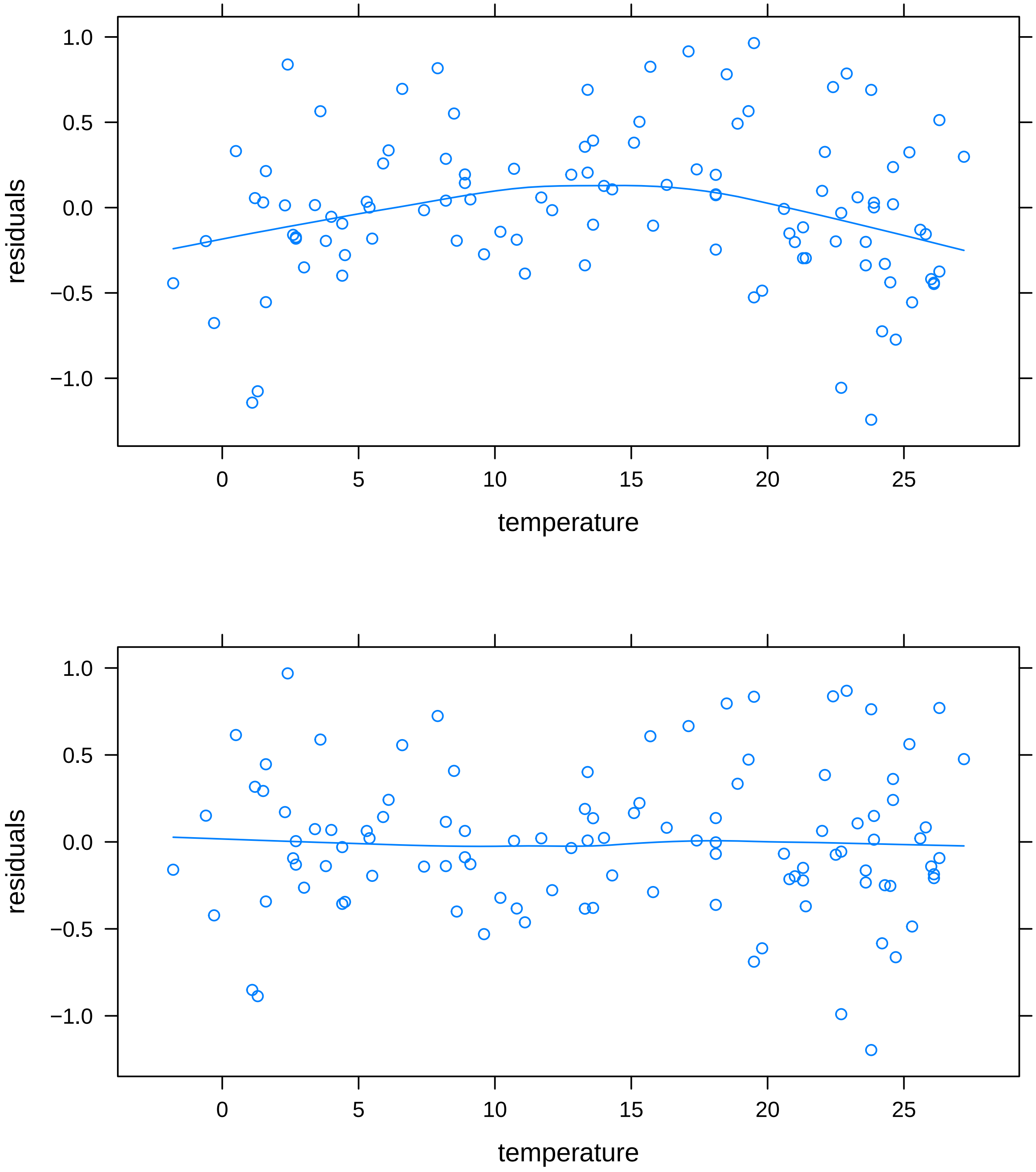

Figure 2: Inclusion of a quadratic term (bottom) eliminates a pattern otherwise found in the residuals from the working model with respect to monthly average temperature (top).

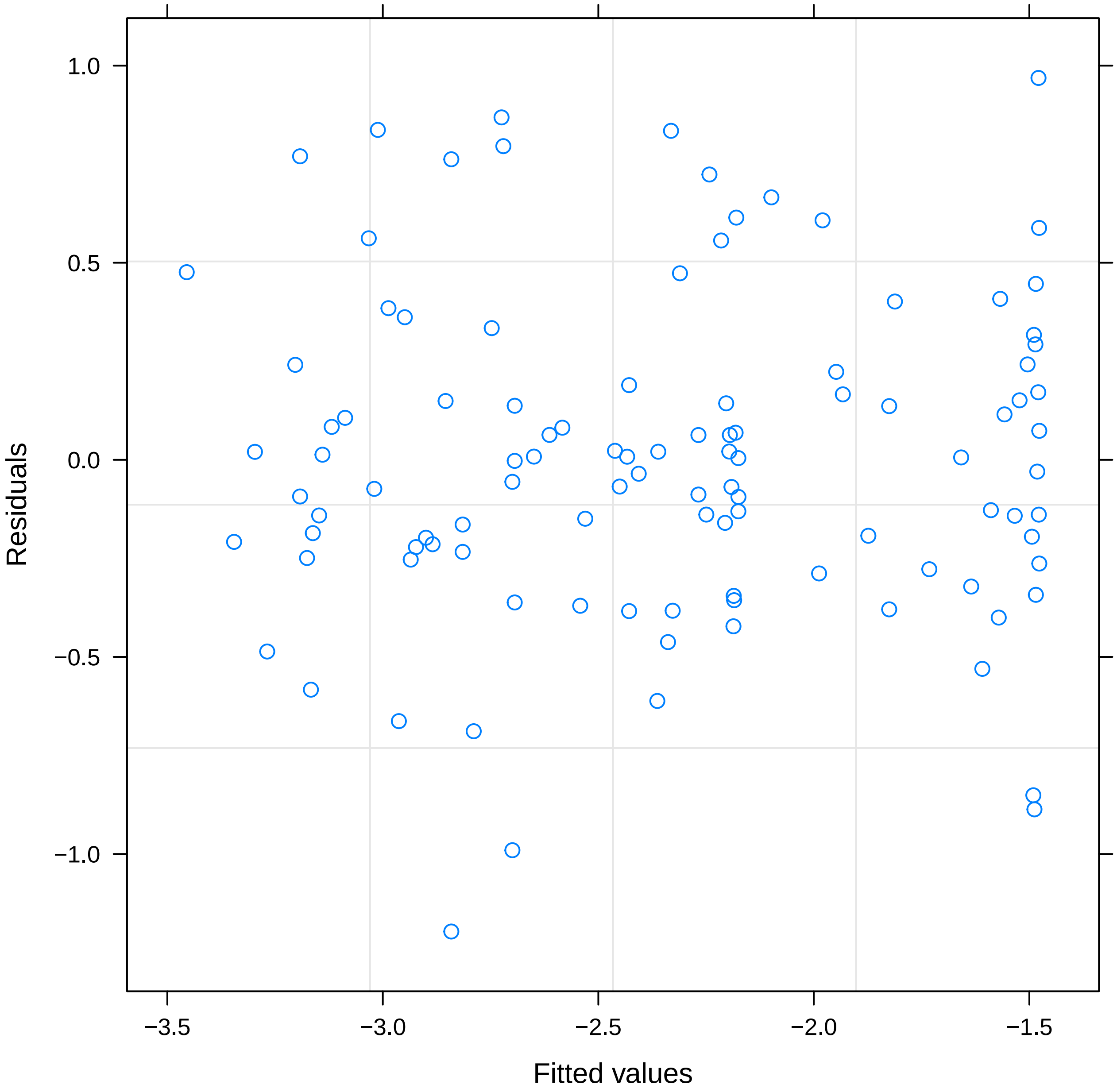

Figure 3: Residuals versus fitted values show no particular pattern that would be concerning.

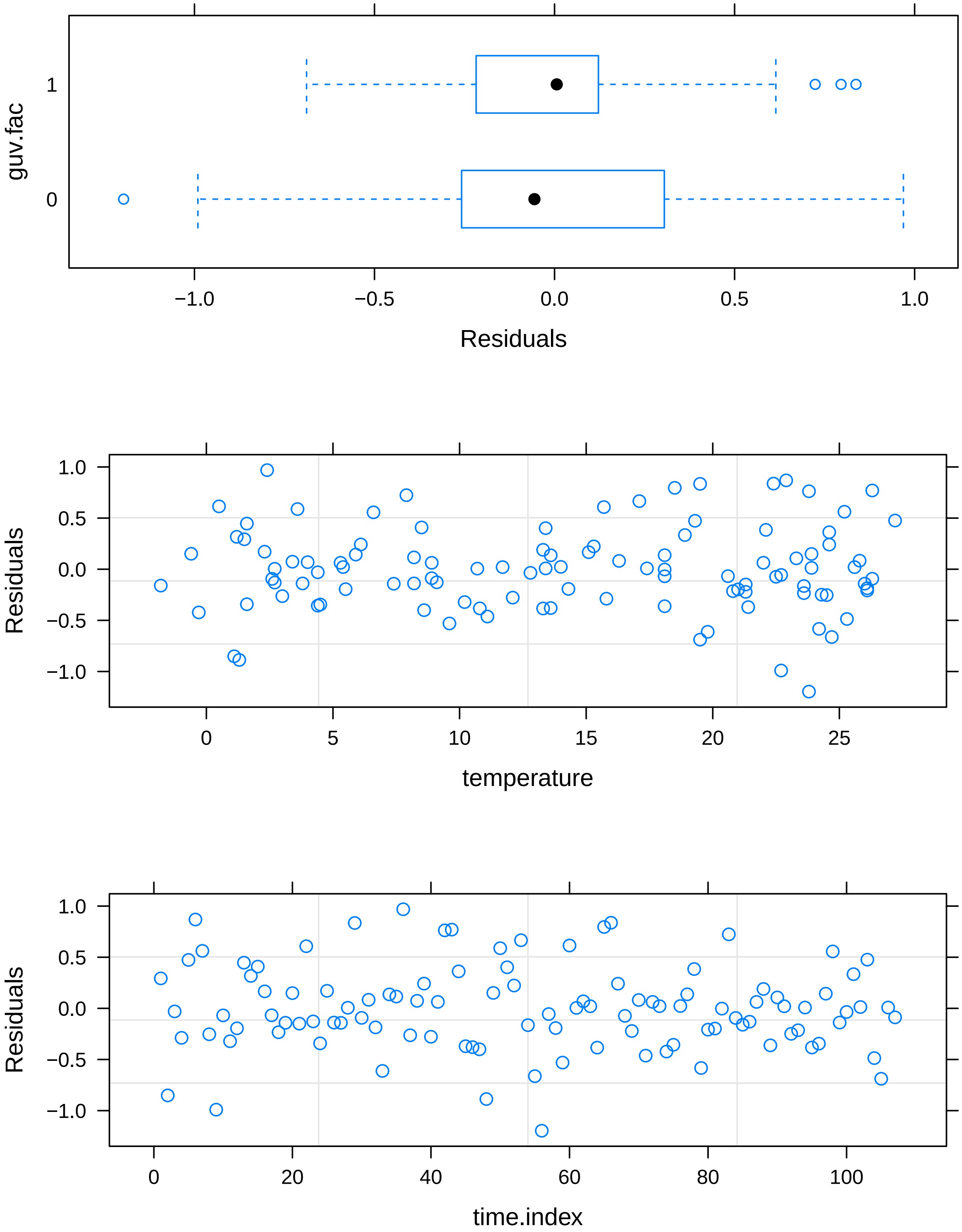

Figure 4: Top: There is slight heteroskedasticity in residuals between pre- and post-GUV periods. Middle: residuals by monthly average temperature show no particular pattern. Bottom: Time participates in the working model only in the serial autocorrelation structure. Residuals by time show no particular pattern.

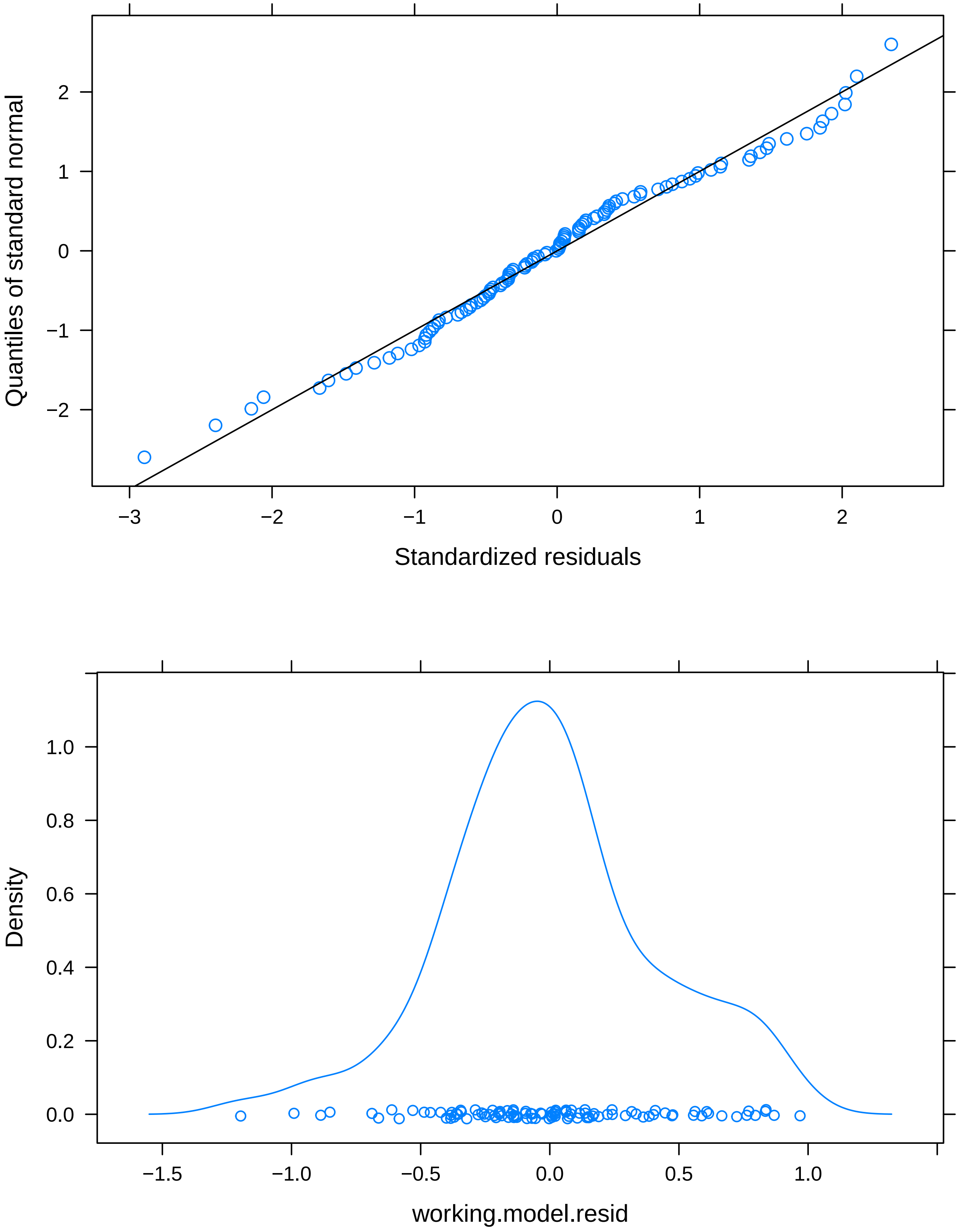

Figure 5: Residuals are a reasonable fit to a Gaussian distribution. Top: qqnormal plot. Bottom: kernel density plot.

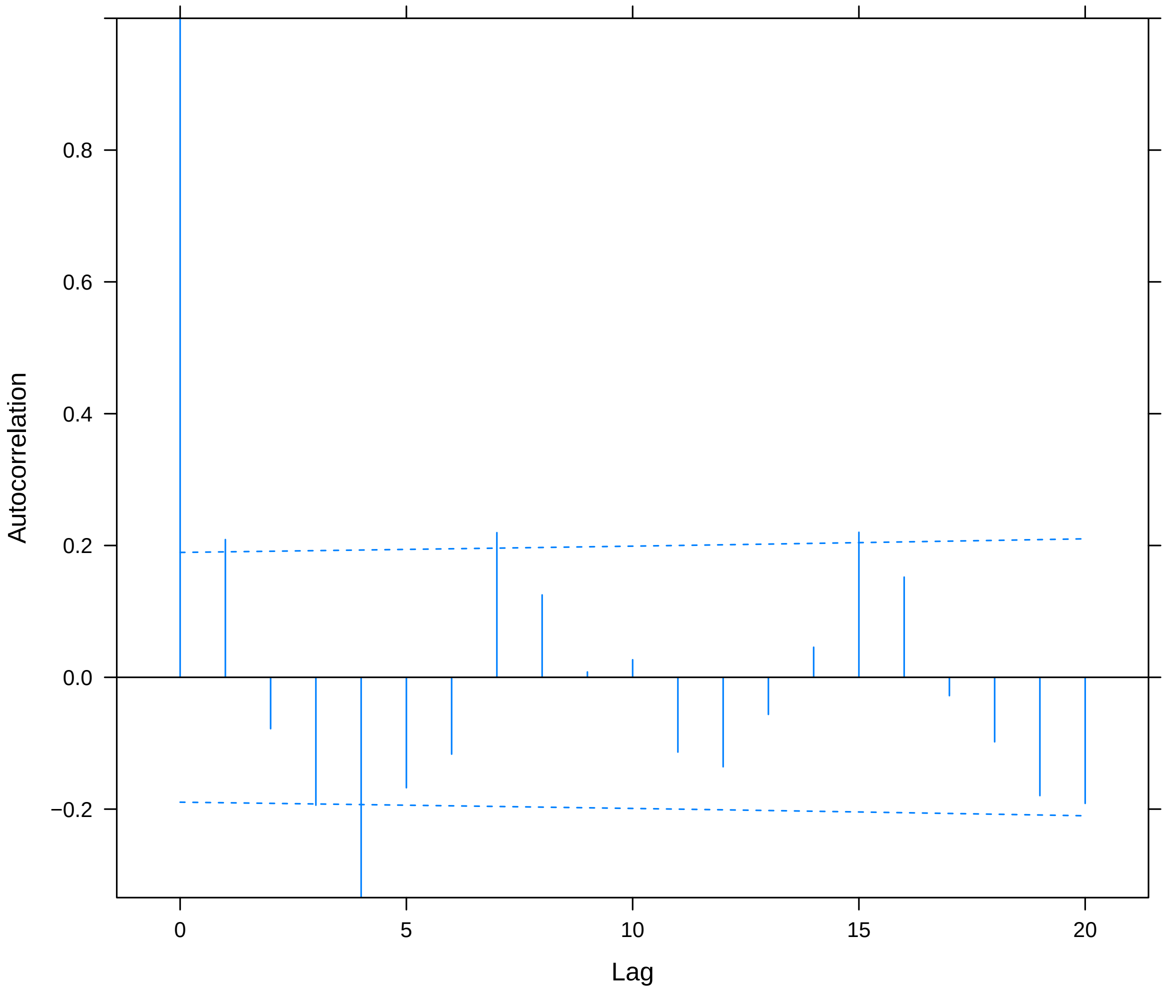

Figure 6: Some residual autocorrelation remains, especially at a lag of four months.

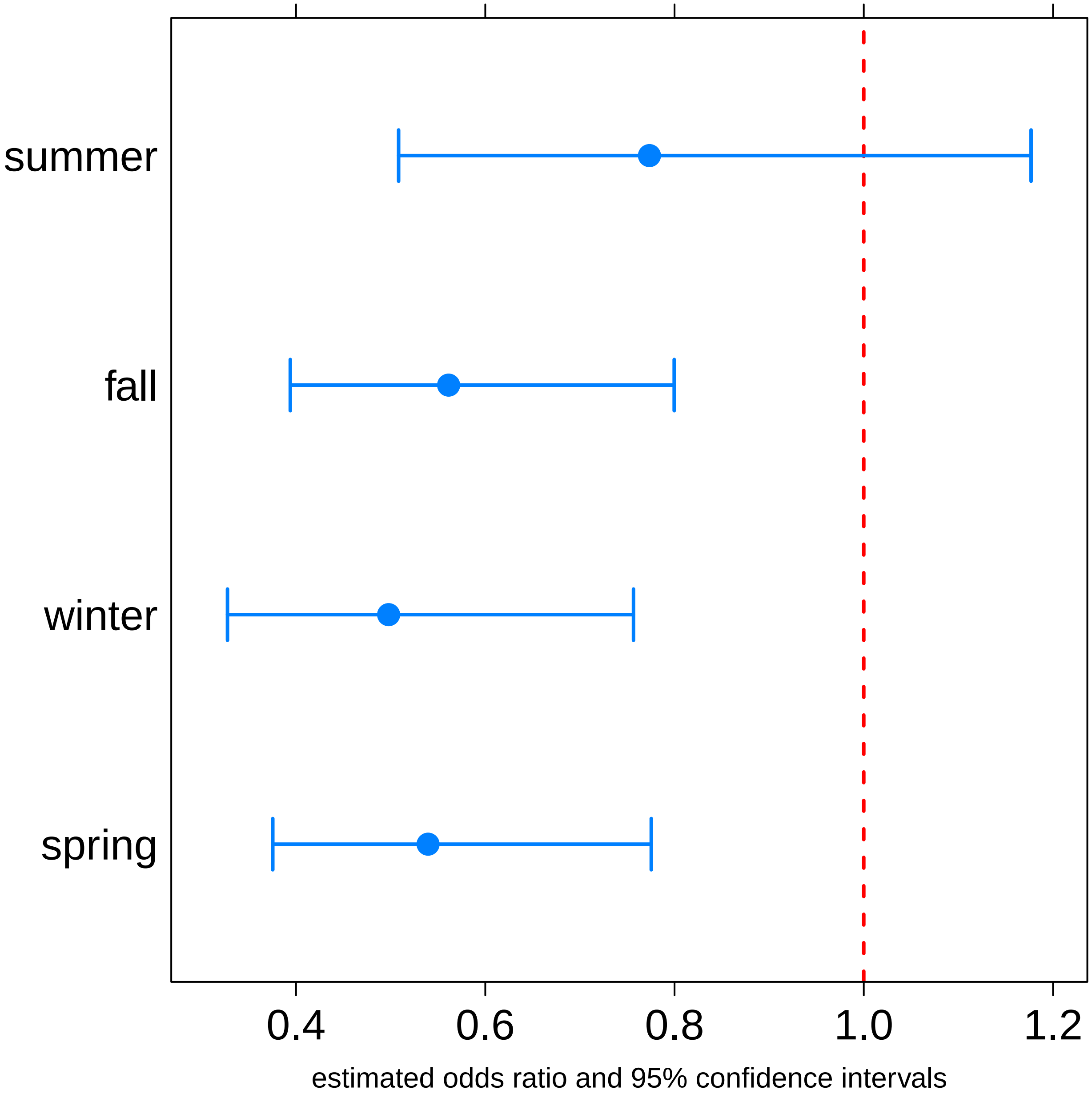

Figure 7: Two-sided odds ratios for effect of GUV on odds of absence, at four illustrative temperatures—the mean temperature for each season.
